# The genome sequence of the Small Phoenix, *Ecliptopera silaceata* (Denis & Schiffermüller, 1775)

**DOI:** 10.12688/wellcomeopenres.19207.1

**Published:** 2023-05-10

**Authors:** Douglas Boyes, Owen T. Lewis, University of Oxford and Wytham Woods Genome Acquisition Lab, Darwin Tree of Life Barcoding collective, Wellcome Sanger Institute Tree of Life programme, Wellcome Sanger Institute Scientific Operations: DNA Pipelines collective, Tree of Life Core Informatics collective, Darwin Tree of Life Consortium

**Affiliations:** 1 UK Centre for Ecology & Hydrology, Wallingford, England, UK; 2 University of Oxford, Oxford, England, UK

**Keywords:** Ecliptopera silaceata, Small Phoenix, genome sequence, chromosomal, Lepidoptera

## Abstract

We present a genome assembly from an individual male *Ecliptopera silaceata* (the Small Phoenix; Arthropoda; Insecta; Lepidoptera; Geometridae). The genome sequence is 316.5 megabases in span. Most of the assembly is scaffolded into 29 chromosomal pseudomolecules, including the assembled Z sex chromosome. The mitochondrial genome has also been assembled and is 17.5 kilobases in length. Gene annotation of this assembly on Ensembl identified 16,770 protein coding genes.

## Species taxonomy

Eukaryota; Metazoa; Ecdysozoa; Arthropoda; Hexapoda; Insecta; Pterygota; Neoptera; Endopterygota; Lepidoptera; Glossata; Ditrysia; Geometroidea; Geometridae; Larentiinae; *Ecliptopera*; *Ecliptopera silaceata* (Denis & Schiffermüller, 1775) (NCBI:txid104457).

## Background

The Small Phoenix, *Ecliptopera silaceata* is a distinctive geometrid moth, the only representative of its genus in Britain and Ireland. There are two main colour forms, differing in the extent to which the broad central forewing band is broken by white markings. The male has a distinctive resting posture, with the tip of its abdomen curled upwards ( Waring *et al.*, 2017).

*Ecliptopera silaceata* is a common species across Britain and Ireland but is absent from Shetland ( Randle *et al.*, 2019). It is found in woodlands and in a wide variety of other habitats, wherever its main larval foodplants, willowherbs ( *Epilobium* spp.), occur ( Waring *et al.*, 2017). This species usually has two and sometimes three generations in the UK, depending on latitude and summer temperatures, and over-winters as a pupa ( Henwood *et al.*, 2020). Its global distribution extends across much of Europe and Asia, as well as parts of northern North America ( GBIF Secretariat, 2022).

A genome assembly for *the Small Phoenix* will contribute to a growing data set of resources for understanding lepidopteran biology. The genome of *E. silaceata* was sequenced as part of the Darwin Tree of Life Project, a collaborative effort to sequence all named eukaryotic species in the Atlantic Archipelago of Britain and Ireland. Here we present a chromosomally complete genome sequence for *Ecliptopera silaceata*, based on one male specimen from Wytham Woods, Oxfordshire, UK.

## Genome sequence report

**Figure 1.**
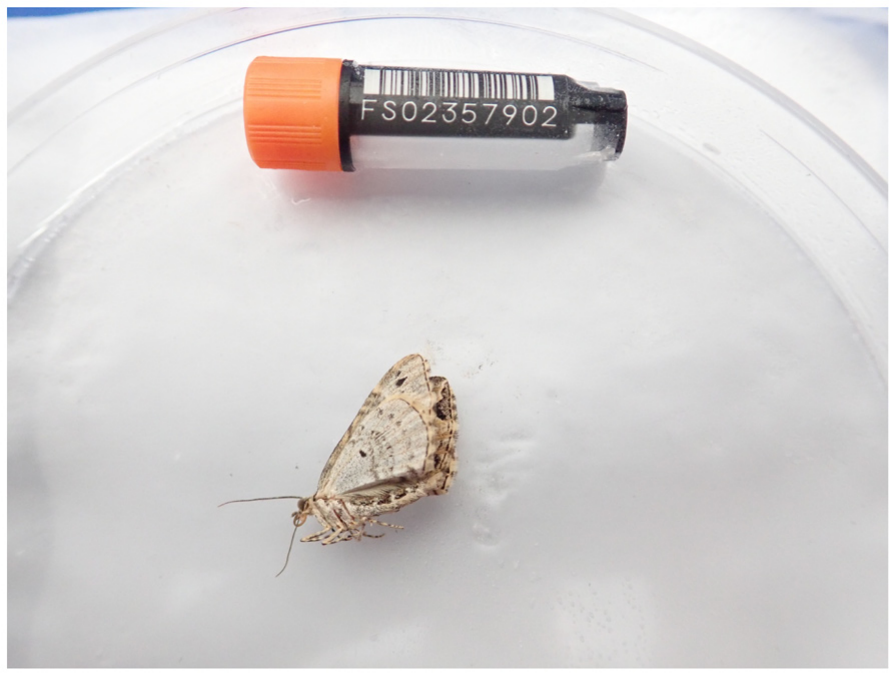
Photograph of the *Ecliptopera silaceata* (ilEclSila1) specimen used for genome sequencing.

The genome was sequenced from one male *E. silaceata* ( Figure 1) collected from Wytham Woods, UK (latitude 51.77, longitude –1.34). A total of 53-fold coverage in Pacific Biosciences single-molecule HiFi long reads and 123-fold coverage in 10X Genomics read clouds were generated. Primary assembly contigs were scaffolded with chromosome conformation Hi-C data. Manual assembly curation corrected 14 missing or mis-joins and removed six haplotypic duplications, reducing the assembly length by 2.47%, and decreasing the scaffold N50 by 0.74%.

**Figure 2.**
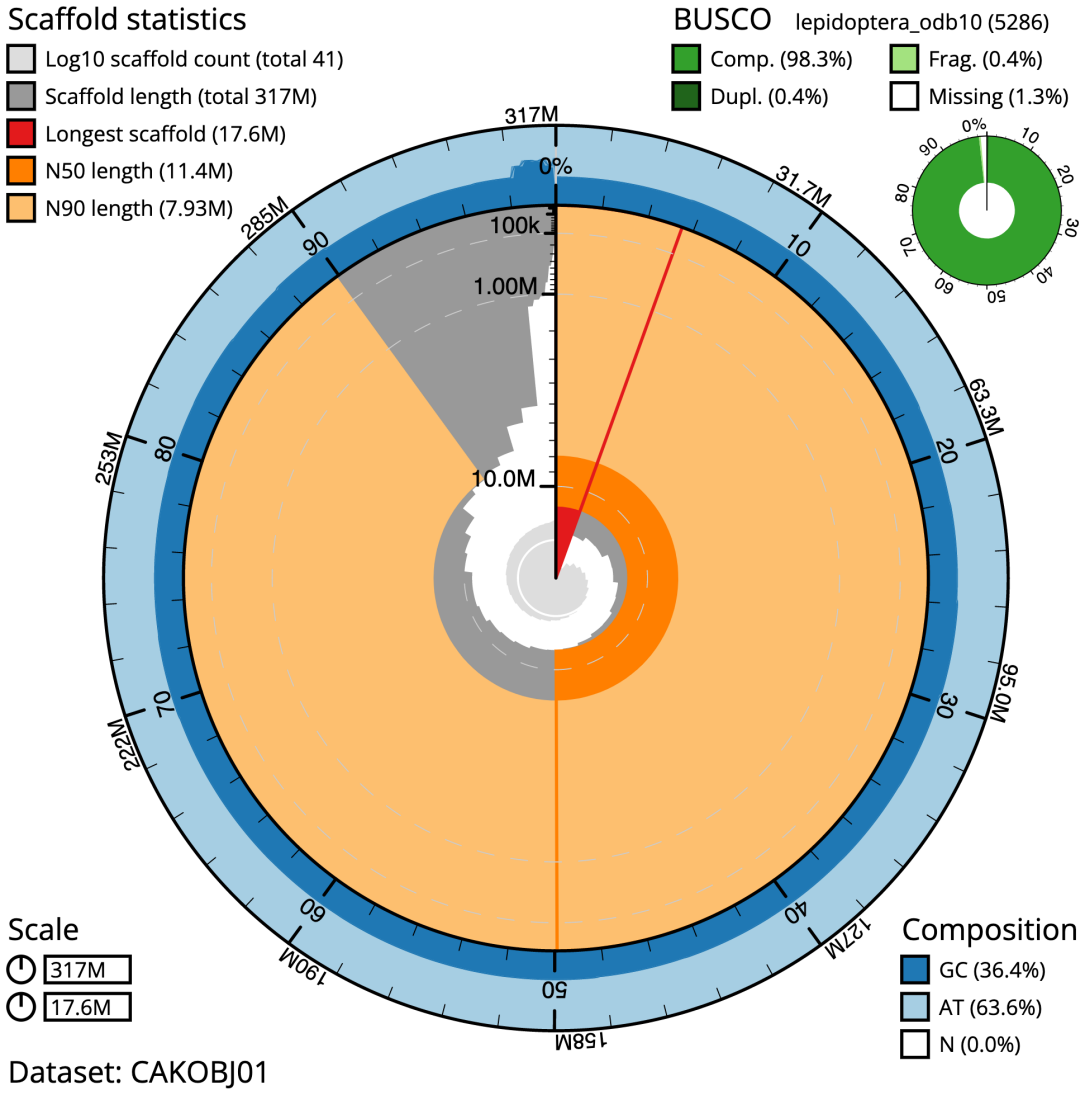
Genome assembly of *Ecliptopera silaceata*, ilEclSila1.1: metrics. The BlobToolKit Snailplot shows N50 metrics and BUSCO gene completeness. The main plot is divided into 1,000 size-ordered bins around the circumference with each bin representing 0.1% of the 316,552,688 bp assembly. The distribution of scaffold lengths is shown in dark grey with the plot radius scaled to the longest scaffold present in the assembly (17,583,276 bp, shown in red). Orange and pale-orange arcs show the N50 and N90 scaffold lengths (11,363,788 and 7,928,850 bp), respectively. The pale grey spiral shows the cumulative scaffold count on a log scale with white scale lines showing successive orders of magnitude. The blue and pale-blue area around the outside of the plot shows the distribution of GC, AT and N percentages in the same bins as the inner plot. A summary of complete, fragmented, duplicated and missing BUSCO genes in the lepidoptera_odb10 set is shown in the top right. An interactive version of this figure is available at https://blobtoolkit.genomehubs.org/view/ilEclSila1.1/dataset/CAKOBJ01/snail.

The final assembly has a total length of 316.5 Mb in 40 sequence scaffolds with a scaffold N50 of 11.4 Mb ( Table 1). Most (98.28%) of the assembly sequence was assigned to 28 chromosomal-level scaffolds, representing 28 autosomes, and the Z sex chromosome. Chromosome-scale scaffolds confirmed by the Hi-C data are named in order of size ( Figure 2– Figure 5; Table 2). The assembly has a BUSCO v5.3.2 ( Manni *et al.*, 2021) completeness of 98.3% (single 97.9%, duplicated 0.4%), using the lepidoptera_odb10 reference set. While not fully phased, the assembly deposited is of one haplotype. Contigs corresponding to the second haplotype have also been deposited.

**Table 1.** Genome data for *Ecliptopera silaceata*, ilEclSila1.1.

Project accession data		
Assembly identifier	ilEclSila1.1	
Species	*Ecliptopera silaceata*	
Specimen	ilEclSila1	
NCBI taxonomy ID	104457	
BioProject	PRJEB50733	
BioSample ID	SAMEA7701534	
Isolate information	ilEclSila1: male; whole organism (PacBio and Chromium) ilEclSila2: head/thorax (Hi-C scaffolding) ilEclSila3: abdomen (RNA-Seq)	
Assembly metrics *		*Benchmark*
Consensus quality (QV)	54.3	*≥ 50*
*k*-mer completeness	99.99%	*≥ 95%*
BUSCO **	C:98.3%[S:97.9%,D:0.4%], F:0.4%,M:1.3%,n:5,286	*C ≥ 95%*
Percentage of assembly mapped to chromosomes	98.28%	*≥ 95%*
Sex chromosomes	Z chromosome	*localised homologous pairs*
Organelles	Mitochondrial genome assembled.	*complete single alleles*
Raw data accessions		
PacificBiosciences SEQUEL II	ERR8575366	
10X Genomics Illumina	ERR8571645–ERR8571648	
Hi-C Illumina	ERR8571656	
PolyA RNA-Seq Illumina	ERR10123668	
Genome assembly		
Assembly accession	GCA_932527185.1	
*Accession of alternate haplotype*	GCA_932527285.1	
Span (Mb)	316.5	
Number of contigs	46	
Contig N50 length (Mb)	11.1	
Number of scaffolds	40	
Scaffold N50 length (Mb)	11.4	
Longest scaffold (Mb)	17.6	
**Genome annotation**		
Number of protein-coding genes	16,770	
Number of non-coding genes	16,974	

* Assembly metric benchmarks are adapted from column VGP-2020 of “Table 1: Proposed standards and metrics for defining genome assembly quality” from ( Rhie *et al.*, 2021). ** BUSCO scores based on the lepidoptera_odb10 BUSCO set using v5.3.2. C = complete [S = single copy, D = duplicated], F = fragmented, M = missing, n = number of orthologues in comparison. A full set of BUSCO scores is available at https://blobtoolkit.genomehubs.org/view/ilEclSila1.1/dataset/CAKOBJ01/busco.

**Figure 3.**
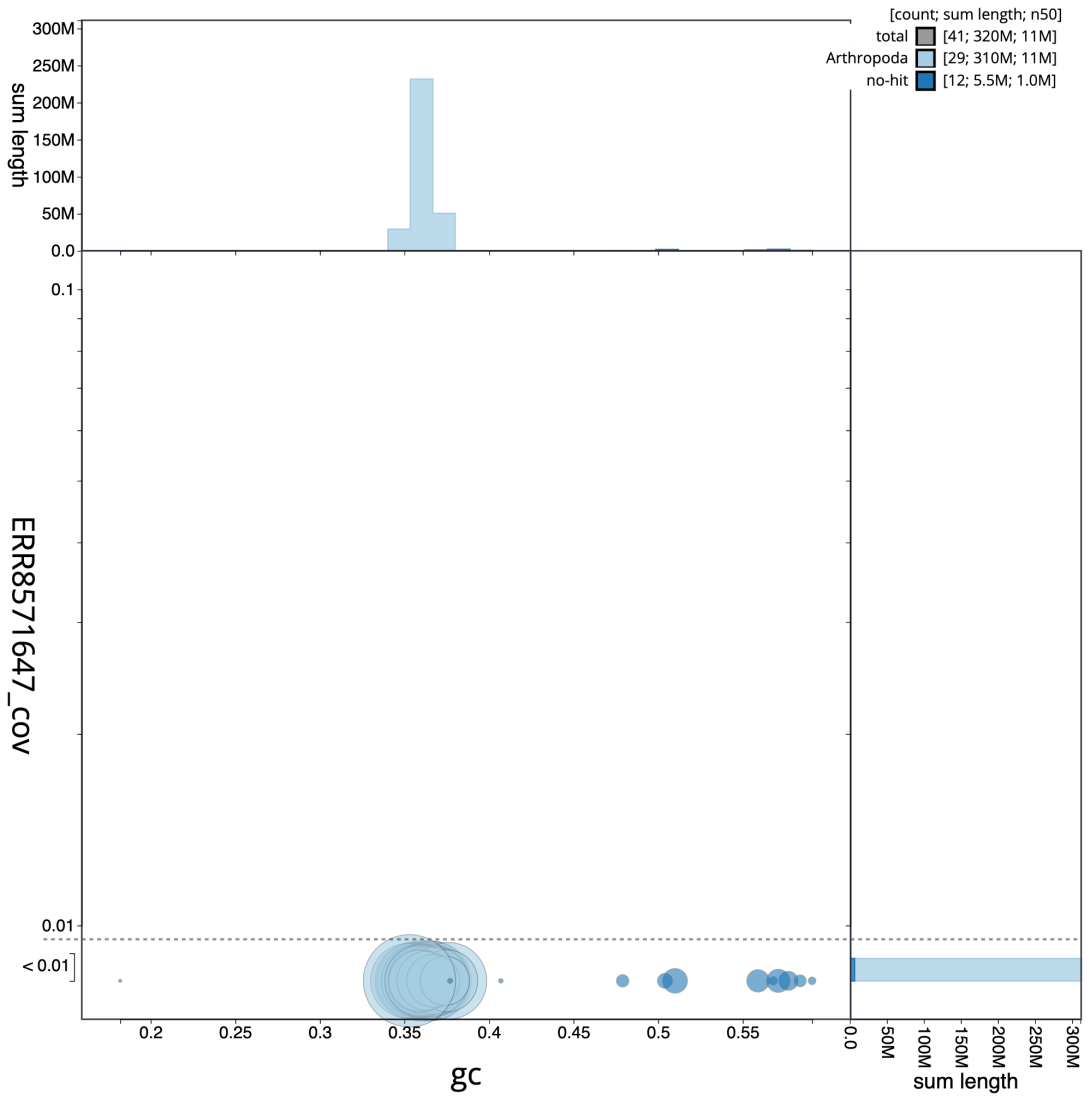
Genome assembly of *Ecliptopera silaceata*, ilEclSila1.1: GC coverage. BlobToolKit GC-coverage plot. Scaffolds are coloured by phylum. Circles are sized in proportion to scaffold length. Histograms show the distribution of scaffold length sum along each axis. An interactive version of this figure is available at https://blobtoolkit.genomehubs.org/view/ilEclSila1.1/dataset/CAKOBJ01.1/blob.

**Figure 4.**
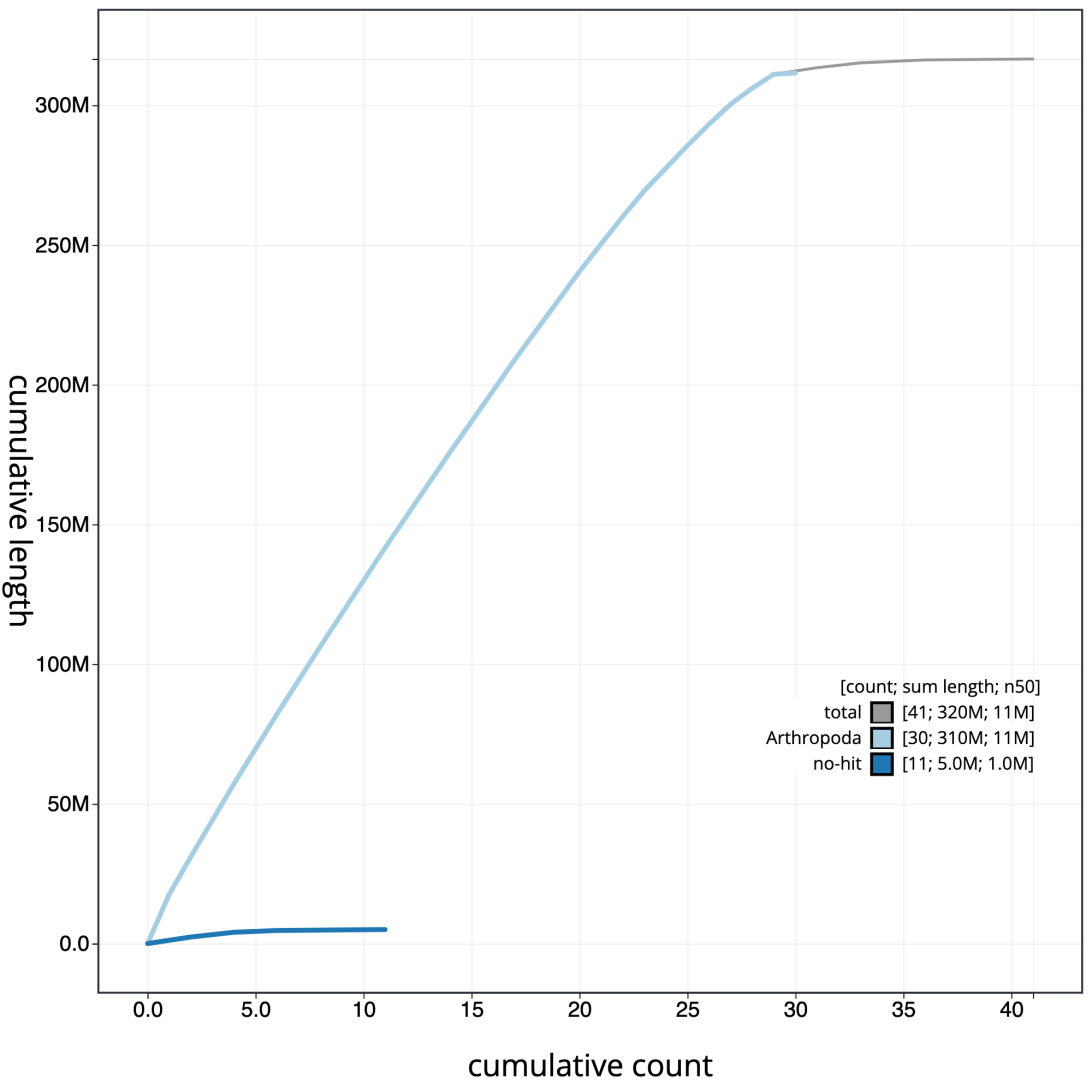
Genome assembly of *Ecliptopera silaceata*, ilEclSila1.1: cumulative sequence. BlobToolKit cumulative sequence plot. The grey line shows cumulative length for all scaffolds. Coloured lines show cumulative lengths of scaffolds assigned to each phylum using the buscogenes taxrule. An interactive version of this figure is available at https://blobtoolkit.genomehubs.org/view/ilEclSila1.1/dataset/CAKOBJ01/cumulative.

**Figure 5.**
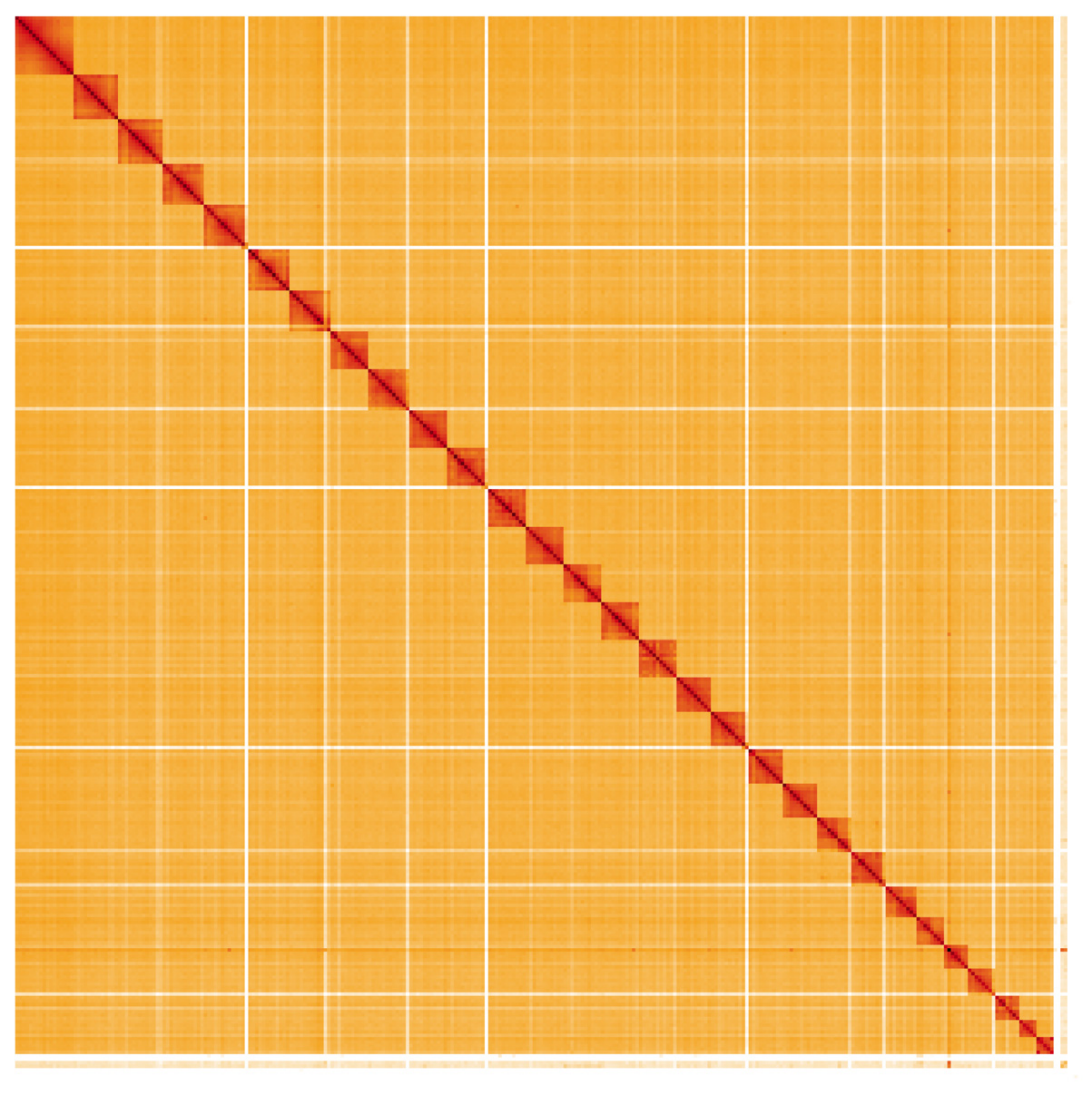
Genome assembly of *Ecliptopera silaceata*, ilEclSila1.1: Hi-C contact map. Hi-C contact map of the ilEclSila1.1 assembly, visualised using HiGlass. Chromosomes are shown in order of size from left to right and top to bottom. An interactive version of this figure may be viewed at https://genome-note-higlass.tol.sanger.ac.uk/l/?d=HbjDrvh-S6yYDYceMQaKUw.

**Table 2.** Chromosomal pseudomolecules in the genome assembly of *Ecliptopera silaceata*, ilEclSila1.

INSDC accession	Chromosome	Size (Mb)	GC%
OW052191.1	1	13.45	36.1
OW052192.1	2	13.08	36.6
OW052193.1	3	13.04	36.5
OW052194.1	4	12.54	35.6
OW052195.1	5	12.51	36.2
OW052196.1	6	12.11	37.6
OW052197.1	7	12.05	36.1
OW052198.1	8	11.83	35.7
OW052199.1	9	11.81	35.5
OW052200.1	10	11.63	35.6
OW052201.1	11	11.45	35.2
OW052202.1	12	11.36	36
OW052203.1	13	11.33	36.1
OW052204.1	14	11.15	36
OW052205.1	15	11.02	35.8
OW052206.1	16	10.92	36
OW052207.1	17	10.64	36
OW052208.1	18	10.49	36.7
OW052209.1	19	10.49	36.1
OW052210.1	20	9.92	35.9
OW052211.1	21	9.72	36.4
OW052212.1	22	9.24	36.8
OW052213.1	23	8.26	35.9
OW052214.1	24	7.93	37.5
OW052215.1	25	7.65	35.9
OW052216.1	26	7.16	36.3
OW052217.1	27	5.73	36.7
OW052218.1	28	4.99	37.4
OW052190.1	Z	17.58	35.3
OW052219.1	MT	0.02	18.3

## Genome annotation report

The *Ecliptopera silaceata* genome assembly (GCA_932527185.1 (ilEclSila1.1) was annotated using the Ensembl rapid annotation pipeline ( Table 1; Ensembl accession number GCA_932527185.1). The resulting annotation includes 16,770 protein-coding and 16,974 non-coding genes.

## Methods

### Sample acquisition and nucleic acid extraction

A male *E. silaceata* (ilEclSila1) was collected from Wytham Woods, Oxfordshire (biological vice-county: Berkshire) (latitude 51.77, longitude –1.34) on 20 July 2020. The specimen was taken from woodland habitat by Douglas Boyes (University of Oxford) using a light trap. Two additional *E. silaceata* specimens (ilEclSila2 and ilEclSila3) were collected by Douglas Boyes from Wytham Woods (latitude 51.77, longitude –1.32) on 28 May 2021. All specimens were identified by Douglas Boyes and preserved on dry ice. The specimen used for DNA sequencing was ilEclSila1, while ilEclSila2 and ilEclSila3 were used for Hi-C scaffolding and RNA sequencing, respectively.

DNA was extracted at the Tree of Life laboratory, Wellcome Sanger Institute (WSI). The ilEclSila1 sample was weighed and dissected on dry ice with tissue set aside for Hi-C sequencing. Whole organism tissue was cryogenically disrupted to a fine powder using a Covaris cryoPREP Automated Dry Pulveriser, receiving multiple impacts. High molecular weight (HMW) DNA was extracted using the Qiagen MagAttract HMW DNA extraction kit. Low molecular weight DNA was removed from a 20-ng aliquot of extracted DNA using the 0.8X AMpure XP purification kit prior to 10X Chromium sequencing; a minimum of 50 ng DNA was submitted for 10X sequencing. HMW DNA was sheared into an average fragment size of 12–20 kb in a Megaruptor 3 system with speed setting 30. Sheared DNA was purified by solid-phase reversible immobilisation using AMPure PB beads with a 1.8X ratio of beads to sample to remove the shorter fragments and concentrate the DNA sample. The concentration of the sheared and purified DNA was assessed using a Nanodrop spectrophotometer and Qubit Fluorometer and Qubit dsDNA High Sensitivity Assay kit. Fragment size distribution was evaluated by running the sample on the FemtoPulse system.

RNA was extracted from abdomen tissue of ilEclSila3 in the Tree of Life Laboratory at the WSI using TRIzol, according to the manufacturer’s instructions. RNA was then eluted in 50 μl RNAse-free water and its concentration assessed using a Nanodrop spectrophotometer and Qubit Fluorometer using the Qubit RNA Broad-Range (BR) Assay kit. Analysis of the integrity of the RNA was done using Agilent RNA 6000 Pico Kit and Eukaryotic Total RNA assay.

### Sequencing

Pacific Biosciences HiFi circular consensus and 10X Genomics read cloud DNA sequencing libraries were constructed according to the manufacturers’ instructions. Poly(A) RNA-Seq libraries were constructed using the NEB Ultra II RNA Library Prep kit. DNA and RNA sequencing were performed by the Scientific Operations core at the WSI on Pacific Biosciences SEQUEL II (HiFi), Illumina NovaSeq 6000 (RNA-Seq and 10X) instruments. Hi-C data were also generated from tissue of ilEclSila2 using the Arima v2 kit and sequenced on the Illumina NovaSeq 6000 instrument.

### Genome assembly

Assembly was carried out with Hifiasm ( Cheng *et al.*, 2021) and haplotypic duplication was identified and removed with purge_dups ( Guan *et al.*, 2020). One round of polishing was performed by aligning 10X Genomics read data to the assembly with Long Ranger ALIGN, calling variants with FreeBayes ( Garrison & Marth, 2012). The assembly was then scaffolded with Hi-C data ( Rao *et al.*, 2014) using YaHS ( Zhou *et al.*, 2023). The assembly was checked for contamination and corrected as described previously ( Howe *et al.*, 2021). Manual curation was performed using HiGlass ( Kerpedjiev *et al.*, 2018) and Pretext ( Harry, 2022). The mitochondrial genome was assembled using MitoHiFi ( Uliano-Silva *et al.*, 2022), which performed annotation using MitoFinder ( Allio *et al.*, 2020). The genome was analysed, and BUSCO scores were generated within the BlobToolKit environment ( Challis *et al.*, 2020). Table 3 contains a list of all software tool versions used, where appropriate.

**Table 3.** Software tools and versions used.

Software tool	Version	Source
BlobToolKit	4.0.7	Challis *et al.*, 2020
FreeBayes	1.3.1-17-gaa2ace8	Garrison & Marth, 2012
Hifiasm	0.15.3	Cheng *et al.*, 2021
HiGlass	1.11.6	Kerpedjiev *et al.*, 2018
Long Ranger ALIGN	2.2.2	https://support.10xgenomics.com/genome-exome/software/pipelines/latest/advanced/other-pipelines
MitoHiFi	2	Uliano-Silva *et al.*, 2022
PretextView	0.2	Harry, 2022
purge_dups	1.2.3	Guan *et al.*, 2020
YaHS	1	Zhou *et al.*, 2023

### Genome annotation

The BRAKER2 pipeline ( Brůna *et al.*, 2021) was used in the default protein mode to generate annotation for the *Ecliptopera silaceata* assembly (GCA_932527185.1). in Ensembl Rapid Release.

### Ethics and compliance issues

The materials that have contributed to this genome note have been supplied by a Darwin Tree of Life Partner. The submission of materials by a Darwin Tree of Life Partner is subject to the Darwin Tree of Life Project Sampling Code of Practice. By agreeing with and signing up to the Sampling Code of Practice, the Darwin Tree of Life Partner agrees they will meet the legal and ethical requirements and standards set out within this document in respect of all samples acquired for, and supplied to, the Darwin Tree of Life Project. All efforts are undertaken to minimise the suffering of animals used for sequencing. Each transfer of samples is further undertaken according to a Research Collaboration Agreement or Material Transfer Agreement entered into by the Darwin Tree of Life Partner, Genome Research Limited (operating as the Wellcome Sanger Institute), and in some circumstances other Darwin Tree of Life collaborators.

## Data Availability

European Nucleotide Archive: *Ecliptopera silaceata* (small phoenix). Accession number PRJEB50733; https://identifiers.org/ena.embl/PRJEB50733 ( Wellcome Sanger Institute, 2022) The genome sequence is released openly for reuse. The *Ecliptopera silaceata* genome sequencing initiative is part of the Darwin Tree of Life (DToL) project. All raw sequence data and the assembly have been deposited in INSDC databases. Raw data and assembly accession identifiers are reported in Table 1.

## Author information

Members of the University of Oxford and Wytham Woods Genome Acquisition Lab are listed here: https://doi.org/10.5281/zenodo.4789928.

Members of the Darwin Tree of Life Barcoding collective are listed here: https://doi.org/10.5281/zenodo.4893703.

Members of the Wellcome Sanger Institute Tree of Life programme are listed here: https://doi.org/10.5281/zenodo.4783585.

Members of Wellcome Sanger Institute Scientific Operations: DNA Pipelines collective are listed here: https://doi.org/10.5281/zenodo.4790455.

Members of the Tree of Life Core Informatics collective are listed here: https://doi.org/10.5281/zenodo.5013541.

Members of the Darwin Tree of Life Consortium are listed here: https://doi.org/10.5281/zenodo.4783558.
