# Supplementary material for: The genome sequence of the Small Phoenix, Ecliptopera silaceata (Denis & Schiffermüller, 1775)
Source: Wellcome Open Res. 2023 May 10;8:209. [Version 1] doi: 10.12688/wellcomeopenres.19207.1 (PMC11535490; doi:10.12688/wellcomeopenres.19207.1)
Supplement: Peer review history

# Reviewer report: Reviewer response for version 1

**DOI:** 10.21956/wellcomeopenres.21289.r104517

**Published:** 2024-11-04

**Authors:** Linda Neaves

**Affiliations:** 1 Australian National University, Canberra, Australian Capital Territory, Australia

This paper presents the chromosome level genome assembly and gene annotation of the small phoenix, *Ecliptopera silaceata*.

 The reason for sequencing the genome of this species is clearly justification and methodology appears appropriate. A couple of minor suggestions to improve clarity. 

 It would be useful to mention if the other samples were also male, if that is known.

 I would suggest adding gEVAL to Table 3, or whichever software were used from Howe et al 2021.

 Minor: There is an unnecessary full stop in " *Ecliptopera silaceata* assembly (GCA_932527185.1). in Ensembl Rapid Release."

Are sufficient details of methods and materials provided to allow replication by others?

Yes

Is the rationale for creating the dataset(s) clearly described?

Yes

Are the datasets clearly presented in a useable and accessible format?

Yes

Are the protocols appropriate and is the work technically sound?

Yes

Reviewer Expertise:

Conservation genomics

I confirm that I have read this submission and believe that I have an appropriate level of expertise to confirm that it is of an acceptable scientific standard.

# Reviewer report: Reviewer response for version 1

**DOI:** 10.21956/wellcomeopenres.21289.r79881

**Published:** 2024-05-03

**Authors:** Chris M Ward

**Affiliations:** 1 Australian Wine Research Institute, Urrbrae, South Australia, Australia

The paper entitled "The genome sequence of the Small Phoenix, *Ecliptopera silaceata* (Denis & Schiffermüller, 1775)" by Boyes *et al*. presents the chromosome level genome assembly and gene annotation of the small phoenix, *Ecliptopera silaceata*.

 I can find no issues with the methodology of the paper. My only suggestion would be to cite the tools used to check for contamination "as described previously ( Howe *et al.*, 2021)". 

**Minor comments:**

 Genome annotation section has an extra period.

Are sufficient details of methods and materials provided to allow replication by others?

Yes

Is the rationale for creating the dataset(s) clearly described?

Yes

Are the datasets clearly presented in a useable and accessible format?

Yes

Are the protocols appropriate and is the work technically sound?

Yes

Reviewer Expertise:

Insect-fungal genomics

I confirm that I have read this submission and believe that I have an appropriate level of expertise to confirm that it is of an acceptable scientific standard.
